# Factors in healthcare violence in care of pregnancy termination cases: A case study

**DOI:** 10.1371/journal.pone.0206083

**Published:** 2018-11-14

**Authors:** Chunxiang Qin, Wei-Ti Chen, Yunlong Deng, Xinchun Liu, Xiaoxia Wu, Mei Sun, Ni Gong, Siyuan Tang

**Affiliations:** 1 Obstetrics Department of the Third Xiangya Hospital & Xiangya School of Nursing, Central South University, Changsha, Hunan, China; 2 University of California, Los Angeles, School of Nursing, Los Angeles, California, United States of America; 3 Department of Clinical Psychology of the Third Xiangya Hospital, Central South University, Changsha, Hunan, China; 4 The Third Xiangya Hospital, Central South University, Changsha, Hunan, China; 5 Xiangya School of Nursing, Central South University, Changsha, Hunan, China; Erasmus Medical Center, NETHERLANDS

## Abstract

**Background:**

Workplace violence is a widely-reported phenomenon among healthcare providers and negatively affects quality of care and treatment. This study aims to understand the potential factors related to HCV through the experiences of women who have undergone a pregnancy termination due to fetal anomaly.

**Methods:**

Qualitative interview was used to collect data in this case study. Forty-one pregnant women who decided to terminate their pregnancy due to fetal anomaly were recruited from four Chinese hospital facilities, including three general hospitals and one specialty hospital in Changsha, Hunan, China. In-depth interviews were conducted from May to September 2017. Content analysis was used to analyze the data.

**Results:**

Several potential factors related to violence in healthcare facilities were identified, including preventive factors, which possibly relieve healthcare violence; and negative experiences, which potentially related to healthcare violence. Preventive factors include healthcare providers gaining patient trust with detailed observation, expressing patient-centered care through discreet behavior, and showing patience and professionalism. Factors related to violence include busy work schedules, hurried visits, mechanized process, patients’ scant medical knowledge and mental distress.

**Conclusions:**

This study highlights potential factors related to healthcare violence. The results will be submitted to the Chinese government’s policy making department in order to improve the healthcare system. We also suggest several important strategies to prevent HCV in a healthcare setting, both in China and globally.

## Introduction

Workplace violence (WPV) is defined by the National Institute for Occupational Safety and Health (NIOSH) [1] as “violent acts (including physical assaults and threats of assaults) directed toward persons at work or on duty” and is categorized as physical assault, physical threat and verbal abuse.[2] WPV toward healthcare providers in healthcare settings has been recognized as a global problem and a major public health concern[2,3]. Studies have reported that 39% - 83.4% of healthcare providers have experienced WPV in different countries including U.S. [2], Germany[4], Japan[5], Greece[6], India[7] and Egypt[8]. Compared to other countries, several cases reported higher rates of WPV experienced by healthcare providers (68%-93%) in China, [9,10,11,12]. Other reports showed that the rate of physical assault was 12.6%[13]. A total of 24 healthcare providers in China (doctors and nurses) died from WPV from 2003–2013 [9]. WPV against healthcare providers in China has attracted worldwide attention [10,12,14]. In this paper, we will use the term healthcare violence (HCV) as WPV occurring in healthcare settings against healthcare providers.

Exposure to HCV has negative physical, psychological, emotional, work functioning, relationship with patients/quality of care, social and financial consequences[15]. Psychological influences (e.g., posttraumatic stress, depression), emotional consequences (e.g. anger, fear) and work functioning impacts (e.g., sick leave, job satisfaction) were described to be the most frequent and significant effects of HCV[15]. Quality of healthcare and treatment was affected by HCV because HCV victims were afraid of their patients and changed their behavior at work[15,16,17]. Some healthcare providers reported that HCV exerted a major impact by increasing their intention to leave their present job [16,17]. Emotional consequences for healthcare providers included feelings of anger, disappointment, stress, fear or humiliation [18]. All these negative effects serve as a reminder that analyzing the risk factors for HCV, in order to develop effective interventions, would be very important in research on HCV.

Chappell and Di Martino (2006) established an interactive model to describe the phenomenon of WPV[19]. In this model, individual risk factors for perpetrating WPV include a history of violence, gender, age, difficult childhood, consumption of alcohol/drugs, mental distress, circumstances that foster violence, difficulties in interpersonal relationships, physical appearance, experience, health, education, personality/temperament, attitude and expectations [19]. Using qualitative[20,21] and quantitative data[6,7,13] collected from healthcare providers, analysis of the causes of HCV found that the main risk factors included mental distress, disease progression and unmet patient expectations (such as patients and their families expecting every patient to recover and survive), patients’ perceptions of using aggressive behavior to seek media attention and avoid paying hospital bills, a lack of implementation of legal provisions to penalize culprits responsible for HCV, long wait times for healthcare, and miscommunication between patients and healthcare providers [2,6,7,13,20,21,22,23]. Factors that resulted in HCV came from organizations, patients and providers alike [24], so the views and experiences of both patients and healthcare providers were important. However, the data collected from patients were limited. One result from a survey evaluating the opinions of patients who admitted to a hospital for any reason, and their relatives, showed that a lack of education and long wait times were connected to HCV [25]. More research into the experiences of patients would be helpful in the analysis of the causes of HCV.

Some studies showed that more cases of HCV were reported from departments of obstetrics and gynecology [26,27]. In this department, pregnant women diagnosed with a fetal anomaly and who went through with termination were more likely to experience high mental distress [28,29]. Additionally, they suffered the diagnosis of fetal anomaly as unexpected (every healthy pregnant woman expects a healthy baby). This suggests that pregnant women diagnosed with a fetal anomaly have several risk factors for perpetrating HCV, their opinions are special importance in analysis of the causes of HCV. And they are a case point because the potential for their violence is greater because of the intensity of their experience, their need for compassionate care, as TOPFA involves deep bereavement and loss[30]. In this paper, we aim to understand the potential factors related to HCV through the experiences of women who have undergone termination of pregnancy due to fetal anomaly (TOPFA).

## Materials and methods

A case study was designed to explore the potential factors related to HCV in significant cases of TOPFA, which may apply and be generalize to others[30]. Qualitative interviews were conducted with women who had undergone a termination procedure due to fetal anomaly at three general hospitals and one specialty hospital in Changsha, Hunan, from May to September, 2017. Obstetricians at these four institutions referred potential participants to the research staff for further discussion about inclusion in the study. Women were recruited at the outpatient clinics of the obstetrics departments when they decided to be admitted for termination. The inclusion criteria were: 1. Pregnant women who had decided to undergo TOP due to a fetal anomaly; 2. Able to write and speak Chinese. Pregnant women with severe complications (e.g., heart failure, severe pre-eclampsia, eclampsia, and/or massive hemorrhage) or diagnosed with a severe mental illness (e.g., psychosis, schizophrenia) were excluded because of the possibility that those issues would affect their accounts of their experiences to researchers. A total of 45 women were identified and invited to participate, but four refused to participate due to personal reasons. The remaining 41 women completed the in-depth interview with a 91% recruitment rate.

### Data collection

All participants received a small reimbursement (50 RMB ≈ 7.5 US Dollar) for their time and effort. In-depth interviews were conducted using a semi-structured interview guide. Generally, the study participants led the discussion, with the interviewers prompting discussion as needed. After communicating with the hospital managers, we obtained permission to use one private room in the inpatient department for our interviews. Individual in-depth interviews were conducted before patient discharge, and audio-recorded in this private room. Each interview took approximately 30–90 minutes and was carried out by a nursing researcher.

### Data analysis

The interviews were then transcribed. Qualitative content analysis was used to gain a deeper understanding, compared to using descriptive analysis alone [31]. The analysis was performed in six steps: (1) The researcher listened to and read through the interviews several times to obtain an impression of all of the material; (2) Meaning units (words, sentences, or paragraphs related to each other through their content and context) were identified; (3) Meaning units were condensed to preserve relevant core expressions; (4) Units were coded and categories were divided into subcategories; (5) Categories were built from the subcategories; (6) After a process of interpretation, focusing on discovering the underlying meanings of the words or the content, categories were united in a comprehensive thematic framework [32]. ATLAS.ti software (ATLAS.ti Scientific Software Development GmbH, Berlin, Germany) was used to code and analyze the data. (Table 1)

**Table 1:** Texts (examples for the first category), codes, categories, and themes of this study*.

Text	Codes	Categories	Themes
……You cannot ask the same question two times because there are so many patients waiting for the care. The doctors said that I have too many persons (patients) a day to give you more time……	Busy; so many patients;	Providers’ busy work schedules influenced care quality	Negative experiences, which potentially related to HCV
……We were waiting in line, the endless line. After waiting for 2–3 hours, the doctors gave us 3–5 minutes……	Endless waiting line		
……Some doctors or nurses are very impatient maybe because they are busy and tired. When you want to ask another question, she was not here. She was caring for another patient……	Impatient; tired		
……	Short visit time; hurried; turned to others quickly; spoke fast; limited time; limited communication; unclear explanation	Hurried visits impacted the quality of communication	
……	Machine; data; few words; feel so cold; assembly line; no empathy	A mechanized diagnostic and treatment process lacked humane care	
……	Helplessness; repeated questions; partly understood; a lack of medical knowledge; complying	Participants’ scant medical knowledge brought feelings of loneliness	
……	Difficult to accept; argued and cried; flared temper; refused to visit healthcare providers; mental distress; hurt	Participants’ mental distress impeded communication	
……	Remembered and described; Observed and asked; noticed and comforted; tried their best	Won trust with detailed observation	Preventive factors, observation that which possibly relieve HCV
……	Repeated comparisons; hesitated; rechecked before reporting	Expressed patient-centered care through discreet behavior	
	Predicted my symptoms; explained slowly; warmly	Showed patience and professionalism	

* These were part of the data from our project about the women of TOPFA. We analysis another part about the management of this group in another manuscript titled “Cognition, emotion, and behaviour in women undergoing pregnancy termination for foetal anomaly: A grounded theory analysis.”

### Ethical approval

The study received approval from the Central South University and Yale University Institutional Review Boards. Pregnant women, who met the inclusion criteria and were interested in participating in the study, met with the research staff; then one of the staff members explained the study, answered questions, and obtained verbal consent.

## Results

Participant mean age was 31.12 years (SD: 5.34 years, range: 22 to 43), while mean gestational age was 23.76 weeks (SD: 2.96 weeks). All participants terminated their pregnancy by surgery. Table 1 provides participant diagnoses. Major themes were extracted from the in-depth interviews: negative experiences, which were potentially related to HCV, included healthcare workers’ busy schedules, hurried visits, lack of a humane care process, and participants’ scant medical knowledge and mental distress. Preventive factors, which could possibly relieve HCV, included healthcare providers gaining trust through detailed observation, expressing patient-centered care through discreet behavior, and showing patience and professionalism coupled with tenderness(Table 2).

**Table 2:** Participants’ diagnoses.

Diagnosis	N = 41(%)
Multiple malformation	11(26.8)
Cardiac malformation	7(17.1)
Cleft lip and palate	7(17.1)
Renal malformations	4(9.8)
Cerebral malformation	2(4.9)
Spine malformation	2(4.9)
Strephenopodia	1(2.4)
Osteogenesis imperfecta	1(2.4)
Duodenal obstruction	1(2.4)
Diaphragmatic hernia	1(2.4)
Severe thalassemia	1(2.4)
Biliary atresia	1(2.4)
Acromphalus	1(2.4)
Genital malformation	1(2.4)

### Negative experiences, potentially related to HCV

#### Challenging work environment and providers’ busy work schedules influenced care quality

Participants expressed their understanding of, and sympathy for healthcare providers’ busy work schedules. Five participants mentioned that their providers were “busy and tired” and noted that it affected the quality of service. In addition to a busy schedule, healthcare providers’ service quality was hindered by a noisy work environment, because there were so many patients waiting. Participants described these conditions in a variety of ways.

“The healthcare providers did not even look at me. They had no time to look at me, even though I just wanted to say ‘Thanks’ before leaving.” (27 years old, diagnosed with fetal cardiac abnormality, 24 gestational weeks)

Some participants quoted comments from friends of hers who were healthcare providers, to show her understanding of the demands of this tiring job. Some participants tried to understand the situation by putting themselves in the healthcare providers’ shoes. However, they still did not accept the quality of the care. When a participant—whose baby, at 25 gestational weeks, was diagnosed with cleft lip and palate—tried to prioritize her needs as a patient, she said, “Objectively, this is understandable. If I was a healthcare worker… [I would be the same.] I mean that I understand it, even if I don’t accept it”.

“The providers are very taciturn when they communicate with us. They only provide the minimum amount of information to prevent HCV, but which can be more irritating as result of a patient’s misunderstanding.” (28 years old, diagnosed with fetal cleft lip and palate, 24 gestational weeks)

#### Hurried visits impacted the quality of communication

“Impatience” became one of the issues causing potential HCV. The participants wanted to see more patience from their healthcare providers. Many participants expressed feeling upset about the “lack of information given by healthcare providers”. Communication was insufficient or ineffective due to the short duration of the healthcare provider visit (three to five minutes). After waiting for two to three hours, participants finally saw their healthcare provider and hoped to receive appropriate management, but the encounter typically lasted a very short time, even when the participants had many unresolved questions, such as questions about detailed information about fetal anomaly, doctor’s explanation or laboratory reports they didn’t understand. Sometimes, participants became very angry when their healthcare provider refused to answer their questions, after they had waited half a day to see the care provider.

“Why? I was asked to leave (said with anger), even though I still had many unanswered questions.” (28 years old, diagnosed with fetal cleft lip and palate, 25 gestational weeks)

A 27-year-old woman, whose baby, at 24-weeks gestational age, had a diagnosed cardiac abnormality, stated that she had used her best communication skills to adapt to the fast pace and obtain effective information in such a short visit.

“So many clients were around the provider. I stood beside her and listened to her suggestions to others. I recorded all my questions on my phone before I went to the hospital. When I met my provider, I asked all the questions in a short time.” (27 years old, diagnosed with fetal cardiac abnormality, 24 gestational weeks)

#### A mechanized diagnostic and treatment process lacked humane care

Participants felt left out when they experienced a mechanized, routinized visit, with little information on their diagnosis and no psychological care provided by their healthcare providers. They stated that their healthcare providers were mainly focused on “data,” “examination,” “medicine,” and “prescription,” during their medical appointments. Participants felt like a “machine” in an “assembly line”, which they described as cold and impersonal.

“That is really an assembly line. When I need to think it over, request a clear answer, I can’t, because [the provider] will never see me again once she starts talking to the next client.” (A participant, 30 years old, diagnosed with fetal chromosome abnormality, 21 gestational weeks)

Humane care was explicitly requested by several participants. One participant recalled that the provider stopped her impatiently and blamed her for asking too many questions. She described feeling agitated, and wondered, “Why is she so busy? Why doesn’t she answer my questions? Were the questions stupid? Why doesn’t she care about me?”

“I think that the providers should be concerned about their patients’ mood, and not just about treating their bodies.” (30 years old, diagnosed with fetal chromosome abnormality, 21 gestational weeks)

#### Participants’ scant medical knowledge brought feelings of loneliness

Participants felt “ignorant” and humiliated about their lack of medical knowledge. They stated that they relied on the healthcare providers’ suggestions. However, in some cases medical science left uncertainty about the diagnosis. Participants expressed helplessness, when they had instead hoped to understand the results of the examinations (such as the ultrasound diagnosis and blood test) or the effects of the treatment during the process of termination. They did not completely understand the medical information, even though it had been explained to them by their healthcare providers. And if they asked the same questions repeatedly, the providers refused to answer, as mentioned previously.

“Doctors and nurses told me that the sensitivity of each person is different. But I still want to know the reason that I am different from others.” (31 years old, diagnosed with fetal renal malformations, 29 gestational weeks)

#### Participants’ mental distress impeded communication

The stress from an uncertain diagnosis combined with a long waiting period to visit an expert contributed to an emotional crisis. Several participants complained about the long wait times they had to sit through before they were able to see a healthcare provider.

“I think everyone would be in a bad mood if they’ve waited for two to three hours to see a healthcare provider.” (28 years old, diagnosed with fetal renal malformations, 25 gestational weeks)

“It is so bothersome that after I waited for two to three hours, the provider gave me three to five minutes.” (Two participants, 28 and 32 years old, diagnosed with fetal cleft lip and palate, at 25 and 26 gestational weeks)

Participants talked about the situation in Chinese clinics, whereby they often waited two to three hours for healthcare providers, and then the providers communicated with them for only three to five minutes. They were also frank in describing their anxious and irritable mood due to the diagnosis of their baby’s anomaly.

“I was anxious when I went to the hospital. I knew that no one had done anything wrong, but I still felt angry, because I was irritable.” (31 years old, diagnosed with fetal renal malformations, 29 gestational weeks)

### Preventive factors for HCV

#### Won trust through detailed observation

Healthcare providers’ encouragement and careful explanation of the prognosis of mothers and babies calmed the study participants. When healthcare providers used appropriate language to describe the details to participants during the treatment process, they were easily able to win patient trust. Women felt they were respected when healthcare providers made eye contact, remembered personal details about them, and praised them for their decision.

“The provider who offered amniocentesis is a good person. He said, ‘I remember you. You came here several times and were very hesitant to get amniocentesis. Your husband is a warm man, but you are a little spoiled by him’.” (22 years old, diagnosed with fetal chromosome abnormality, 23 gestational weeks)

As they were injecting lethal medicine into the amniotic cavity, some providers would respond to the distress they observed in the participants by being supportive and sharing observations from their practice. The participants sensed that these providers were empathizing with them, and they calmed down. For example, a participant (41 years old, diagnosed with fetal chromosome abnormality, 21 gestational weeks) said that she had a closer feeling with a nurse who visited her and told her a few reassuring stories about other women when she was sad and tearful.

## Expressed patient-centered care through discreet behavior

Participants felt they were under good care from responsible providers who could provide a potential solution to their condition. When dealing with a distressed participant, the providers’ discreet treatment could create a warm feeling. In one example, the night shift doctors and nurses came to the participant to explain what might happen during the process, and asked if there were any concerns or questions. In another sample, an obstetrician checked repeatedly on the participant’s condition before reporting the results, and took care to discuss the situation with the participant to decrease any painful feelings she might have been experiencing. Providers’ thoughtful manner made these participants feel safe.

“The provider who did the ultrasound examination was very careful. I think he knew there was a fetal abnormality as soon as he began to examine me. But he examined me for a very long time. When I asked him about the condition of the fetus, he said that he needed to check and check and check again. And then, he invited me to another room and said, “I don’t know how to say it when this kind of thing happens…” and so he gave me a sign that I needed to prepare myself for bad news. I think he was not only telling me the result, but also giving me time to prepare myself psychologically.” (37 years old, diagnosed with fetal cleft lip and palate, 24 gestational weeks)

## Showed patience and professionalism

Patience is one of the keys to enhancing the patient-provider relationship. After slowing their pace, providers had time to explain their management plan and showed a high level of professionalism. Some participants evaluated the quality of their healthcare providers only based on their patience.

“My examination at the county hospital impressed me. There were fewer patients at that hospital, and the healthcare providers took time to explain things to me very slowly and completely.” (27 years old, diagnosed with fetal cardiac abnormality, 24 gestational weeks)

In the Chinese medical system, a specialist, who is applied by a senior physician and permitted by the professorial committee in a tertiary hospital according to their professional level, provides care at both a specialty and a general clinic. The specialist in the specialty clinic can explain and discuss the patient’s condition in greater detail during a longer visit (giving each patient more than half an hour at a time) than at a general clinic. Patients (five participants in this study) who can afford it and are willing to pay extra can make an appointment with the specialist in the specialty clinic (the price of the specialty clinic was four times that of the general clinic). These clients can then obtain a more thorough and individually tailored intervention. The experiences with the specialist impressed these participants, who stated that these visits were more detailed and provided them with a great deal of satisfaction.

“Perhaps all patients in China want to receive care provided by specialists in special clinic. Specialists are tender and marvelous. Sometimes it seems like they already know your symptoms before you even ask questions, or they can give you information on exactly what you need.” (27 years old, diagnosed with fetal chromosome abnormality, 21 gestational weeks)

## Discussion

In this paper, we explore pregnant women’s experiences as they went through a termination decision after a fetal anomaly diagnosis. Our results showed two kinds of experiences, including preventive factors and negative experiences.

### Study significance

The main strength of this study was its exploration of the experiences of women terminating their pregnancy due to fetal anomaly, and its exploration of the issues related to HCV in China [9,33,34]. When policy makers and researchers focus on interventions to prevent HCV, patient experiences are important, since patients are the main group (76%) perpetrating HCV [2].

The second strength was that we recruited women who terminated their pregnancy due to fetal anomaly as participants. They should be considered a group with several risk factors for perpetrating HCV, according to Chappell and Di Martino’s interactive model [19]. We collected the experiences of these women while they were seeking obstetrical care to provide baseline data for future effective interventions to prevent HCV.

The third strength was that triangulation was used to facilitate the validation. Memos were written with supporting data to describe each of the codes and themes. The validation of all steps was carefully considered by the research team. Two researchers (Qin and Li) independently checked the analysis from steps two to six and discussed their findings with the research group several times before reaching a final agreement.

### Waiting times and visiting times with insufficient communication and a lack of humane care were the main issues

According to our results, participants stated that long wait times to see their doctor (two to three hours on average) and their doctor having very little time (three to five minutes) to spend with them, were their top concerns. Their doctor’s limited time for seeing patients was often accompanied by impatience on the part of the healthcare provider. These results were consistent with other studies[6,20], which suggested that in most Chinese hospitals, doctors typically see 30 or more patients within four hours [35]. Chinese healthcare providers must complete each visit within three to five minutes, because there are too many patients waiting for them. The participants in our study stated that hurried visits impacted the quality of communication, and an “assembly line” diagnostic and treatment process lacked humane care. Pompeii et al. also noted that patient unhappiness with care, including long waits for care, was one of the factors in perpetrating HCV events [2]. Giving more time for each patient was advocated by the participants in our study.

### Lack of medical knowledge and mental distress were other issues

Participants were eager to learn more about their condition, and their limited medical knowledge made them feel ignorant and humiliated. In Yasar and team’s study (2017) [25], lower education levels among patients and their relatives were found to be related to increased HCV. If limited medical knowledge is related with lower education levels, this is an area that needs to be explored in the future. Participants’ eagerness for medical knowledge is also a reminder to the Chinese government that different methods (such as providing healthcare information online) to share medical knowledge with the public is required. Our participants frankly expressed their distress and irritated emotions, which were aggravated when they waited a long time to see their healthcare providers, and influenced by the providers’ attitude and the work environment. This was the same as the description in Chappell and Di Martino’s model, which noted that mental distress was a risk factor for HCV. [19].

### Communication skills intervention would benefit the patient-provider relationship

Our results also show that appropriate language to describe participant details, and discreet behavior on the part of healthcare providers, made participants feel better, even when the providers didn’t have sufficient time to provide thorough care. Communication skills intervention was proven to be an effective strategy for reducing HCV. [36,37]. Understanding individuals’ needs is a goal of healthcare providers in the care they provide[38]. Engaging healthcare providers in a training program with diverse first-person experience care had been reported as an effective training strategy [38]. The experience of participants in the current study provides important information to ensure that effective communication should be established to reduce HCV.

Interestingly, one of our participants described the special skills that she developed to obtain the maximum amount of information from healthcare workers in a limited amount of time. Maclachlan et al. (2016) developed and implemented a “Patient Empowerment” training curriculum to improve the quality of patient-provider interactions, which uses a similar strategy. [39]. In response to the strained relationship between healthcare providers and patients in China, Xu et al. (2013) introduced a new concept called “nice patient” [40]. They described a model of a desirable patient, who would recognize the uncertainty of medicine and who would collaborate with healthcare providers on their health. Our findings provided suggestions to improve the training curriculum for patients to include communication skills during visits with their healthcare provider.

### Limitations

There were several limitations in this study: First, we recruited our participants in one city, so the generalizability of the findings is limited. Second, we did not recruit healthcare providers into this study. Healthcare providers might have had different experiences of healthcare violence. Finally, families were not recruited. Therefore, we do not know whether their family members had experienced a similar situation, and whether they could confirm the patient's experiences.

## Conclusion

Through the lens of the experiences of pregnant women diagnosed with a fetal abnormality, this study reminds us that providers’ busy work schedules, hurried visits in general clinics, a mechanized diagnostic and treatment process, participants’ scant medical knowledge and mental distress were all negative experiences that related to HCV. The study findings will be submitted to the Chinese government and policy making department to improve the healthcare system. This study also suggests several important strategies to prevent HCV in a healthcare setting, both in China and globally.
